# The Fimbrial Gene *z3276* in Enterohemorrhagic *Escherichia coli* O157:H7 Contributes to Bacterial Pathogenicity

**DOI:** 10.3389/fmicb.2018.01628

**Published:** 2018-07-19

**Authors:** Bicheng Zhang, Xiaohan Sun, Hongjie Fan, Kongwang He, Xuehan Zhang

**Affiliations:** ^1^Institute of Veterinary Medicine, Jiangsu Academy of Agricultural Sciences, Nanjing, China; ^2^Key Laboratory of Engineering Research of Veterinary Bio-products of Agricultural Ministry, Nanjing, China; ^3^National Center for Engineering Research of Veterinary Bio-products, Nanjing, China; ^4^Jiangsu Key Laboratory for Food Quality and Safety-State Key Laboratory Cultivation Base of Ministry of Science and Technology, Nanjing, China; ^5^Jiangsu Co-innovation Center for Prevention and Control of Important Animal Infectious Diseases and Zoonoses, Yangzhou University, Yangzhou, China; ^6^College of Veterinary Medicine, Nanjing Agricultural University, Nanjing, China

**Keywords:** EHEC O157:H7, genetic marker, *z3276*, motility, biofilm formation, invasion, pathogenicity

## Abstract

Enterohemorrhagic *Escherichia coli* (EHEC) O157:H7 is a zoonotic pathogen of worldwide importance that causes foodborne infections in humans. It is not capable of expressing type I fimbrial because of base deletion in the *fim* operon. BLAST analysis shows that the open reading frame *z3276*, a specific genetic marker of EHEC O157:H7, encodes a sequence with high amino acid identity to other *E. coli* type I fimbrial, but its definitive function in EHEC O157:H7 remains unclear. We are here to report that a *z3276* mutant (Δ*z3276*) was constructed using the reference EHEC O157:H7, the mutant Δ*z3276* was biologically characterized, and the pathogenicity of Δ*z3276* was assessed in mice in comparison with the wild-type (WT) strain. Motility and biofilm formation assays revealed a smaller twitching motility zone for Δ*z3276* on the agar surface and significantly decreased biofilm formation by Δ*z3276* compared with the parental strain. The adhesion and invasion ability of Δ*z3276* to HEp-2 cells showed no significant change, but the invasion ability of Δ*z3276* to IPEC-J2 cells was attenuated. Furthermore, in the animal study, we observed shortened and lower fecal shedding among the Δ*z3276* mutant-infected animals compared with those infected WT strain. The data in this study indicate that this unique gene of *z3276* in EHEC O157:H7 seems to play an important role in bacterial pathogenicity.

## Introduction

Since first being recognized in 1982 following an outbreak of hemorrhagic colitis in the United States (Riley et al., 1983), Enterohemorrhagic *Escherichia coli* (EHEC) O157:H7 has emerged as a significant cause of serious human gastrointestinal disease worldwide (Tarr et al., 2005). The clinical manifestations of EHEC O157:H7 infections range from self-limiting diarrhea to hemorrhagic colitis, which can lead to severe complications known as hemolytic uremic syndrome, which is associated with a mortality rate of 2–10% (Kaper et al., 2004; Tarr et al., 2005; Makvana and Krilov, 2015).

Multiple fimbrials and fimbrial gene clusters have been implicated in contributing to the adherence of EHEC O157:H7 to host cells and its virulence (Li et al., 1997; Low et al., 2006; Lloyd et al., 2012; Russell et al., 2018). The hemorrhagic coli pilus of EHEC O157:H7, a type IV pilus, has been associated with intestinal adherence and invasion and can induce proinflammatory cytokine secretion in intestinal epithelial cells (Xicohtencatl-Cortes et al., 2007; Ledesma et al., 2010; Mazariego-Espinosa et al., 2010). Type I fimbrial, composite surface fibers present in various types of pathogenic *E. coli* (Uropathogenic *E. coli* and Diffusely adherent *E. coli*), are important bacterial adhesion organelles present in most Gram-negative bacterial strains that facilitate bacterial colonization (Epler Barbercheck et al., 2018). These fimbrial are encoded by the *fim* gene cluster and are exported by the chaperone/usher pathway, in which FimC is the periplasmic chaperone, FimD is the outer membrane usher, and FimA and FimH are the major structure for adhesion (Epler Barbercheck et al., 2018; Russell et al., 2018). However, EHEC O157:H7 is not able to express type I fimbrial despite containing the *fim* operon because of a 16-bp deletion at the 5’ end of *fim*A and a C to A mutation at position 467 in the *fim*H gene (Li et al., 1997; Low et al., 2006). A 1035-bp open reading frame (ORF), named *z3276*, has been firstly reported in the EHEC O157:H7 genome (Perna et al., 2001). The bioinformatics studies indicated that the *z3276* was properly unique in the genome and identified as a specific marker of EHEC O157:H7 (Perna et al., 2001; Ravan and Amandadi, 2015). It was used to successfully developed detection method, i.e., real-time PCR (Li and Chen, 2012; Li et al., 2017). The protein encoded by the *z3276* gene shows identity to type I fimbrial, but its definitive function in EHEC O157:H7 remains unclear.

To investigate the function of Z3276, a mutant (Δ*z3276*) of EHEC O157:H7 with the *z3276* gene deleted was constructed. Our results suggested that *z3276* played important roles in the bacterial motility, biofilm formation, invasion of specific cell types and colonization *in vivo* of EHEC O157:H7.

## Materials and Methods

### Bacterial Strains, Plasmids, and Growth Conditions

Bacterial strains and plasmids involved in this study are presented in **Table 1**. *E. coli* C118 and SM10 strains were used to prepare the complement cells for pMEG375 and its recombinant plasmids. Theses *E. coli* strains were routinely grown in Luria-Bertani (LB) broth or on LB agar at 37°C, whereas the *E. coli* EDL933 parent strain, the Δz3276 mutant strain, and the complement strain (cΔ*z3276*) were cultured in tryptone soya broth (TSB) or on tryptic-soy agar (TSA) at 37°C to prepare bacterial cultures for comparative adherence assays, invasion assay and twitching motility assay. When required, the antibiotics kanamycin (100 μg/mL), ampicillin (100 μg/mL), chloramphenicol (34 μg/mL) and/or gentamicin (50 μg/mL) were added to the media.

**Table 1:** Bacterial strains and plasmids used in this study.

	Genotypes and phenotypes	Sources or reference
**Strains**		
EDL933	Wild-type EHEC O157:H7 EDL933 strain, SmR	Current lab
BL21 (DE3)	Host for expressing recombinant plasmid	TaKaRa
Trans5α	Host for cloning recombinant plasmid	TaKaRa
CC118	Δ(ara-leu) araD Δlac X74 ga1K phoA20 thi-1 rpsE rpoB argE(Am) recA1, λpir	Current lab
SM10	thi-1 thr leu tonA lacY supE recA::RP4-2-Tc:Mu, KmR, λpir	Current lab
CC118-1	CC118 (pMEG375-*z3276*FRGm), CmR	This work
SM10-1	SM10 (pMEG375-*z3276*FRGm), SmS, GmR, CmR	This work
Δ*z3276*	Stable Δ*z3276* mutant, SmR, GmR, CmS	This work
cΔ*z3276*	Stable CΔ*z3276* mutant, SmR, GmR, ApR	This work
**Plasmids**		
pMEG375	sacRB mobRP4 oriR6K, CmR, ApR	Current lab
pMEG375-z3276FRGm	pMEG-375:: Δ*z3276*:: Gm, CmR	This work
pFastBac	ApR, GmR	Current lab
pMD19-T	ApR	TaKaRa
pMD19-T-*z3276*	ApR	This work
pCold I	ApR	TaKaRa
pCold I-*z3276*	ApR	This work

### Cell Line Culture Conditions

To investigate the Z3276 role in the adherence and invasion to non-intestinal cells and intestinal cells, perhaps by recognition of a common surface receptor, we employed human laryngeal carcinoma cell line HEp-2 (BioVector NTCC Inc.) and porcine neonatal jejunal epithelial cell line IPEC-J2 (a gift from Dr. Zhu Guoqiang, Yangzhou University). These cells were cultured in antibiotic-free RPMI1640 medium, supplemented with 10% newborn calf serum (Sigma-Aldrich) at 37°C in a humidified incubator in an atmosphere of 5% CO_2_.

### Phylogeny Analysis of the Z2376 Amino-Acid Sequence

To explore the homology of Z2376 protein in the protein database of GenBank, its amino-acid sequence was submitted to GenBank to identify its phylogenetic relationships and conserved domain. The phylogenetic tree was generated by BLAST analysis.

### Antigen Cloning, Expression, and Purification

The *z3276* ORF is between 2,951,428 and 2,952,462 nucleotide of EHEC O157:H7 complete genome (GenBank, CP015855.1), far from the disabled *fim* operon, located in the 5,444,749 nucleotide. The PCR product of the complete ORF of *z3276* with primers (P6-F/R) were amplified from chromosomal EHEC O157:H7 and introduced into pCold I to generate recombinant bacteria BL21 (DE3)/pCold I-*z3276.* The recombinant bacterium was grown overnight, subcultured into fresh medium, and further grown for 2 h at 37°C; and isopropyl β-d-thiogalactopyranoside (IPTG) was added and incubation was continued for 24 h. The bacterial cultures were harvested by centrifugation and resuspended in PBS, containing 1 mM Pefabloc, 0.5 mg/mL lysozyme, 10 μg/mL DNase I, and 10 μg/mL RNase A. Cell lysates were ultrasonicated for 5 min with 30 s intervals on ice. Centrifuged supernatants were purified using His∙Bind Resin Chromatography according to the manufacturer’s instructions (GE Healthcare Life Sciences).

### Polyclonal Antibody Preparation

For anti-recombinant Z3276 protein polyclonal antibody preparation, Jiangsu Academy of Agricultural Sciences Institutional Animal Care and Use Committee approved the animal procedures (SYXK2015-0066) in the context of the guidelines of the Jiangsu Province Animal Regulations (Government Decree No. 45). New Zealand white rabbits were obtained from Jinling rabbit Farm (Nanjing, China), housed in cages (W 50 cm, L 30 cm, H 40 cm). They were provided with food and water *ad libitum*. At the end of the study, they were euthanized by intravenous injection of barbiturate, exsanguinated, and blood was clotted at 37°C for 0.5 h, chilled at 4°C for overnight. The purified recombinant protein Z3276 was used as antigen for polyclonal antibody preparation. Polyclonal antibody against Z3276 protein was obtained by immunizing New Zealand White rabbits subcutaneously at multiple sites with approximately 0.5 mg of purified protein emulsified 1:1 in Freund’s complete adjuvant. The rabbits received one booster injection with the same antigen concentration emulsified 1:1 with Freund’s incomplete adjuvant 14 days later and then were bled 10 days after the booster was administered. Sera were stored in -80°C freezer.

For anti-EHEC O157:H7 polyclonal antibody preparation, Jiangsu Academy of Agricultural Sciences Institutional Animal Care and Use Committee approved the animal procedures (SYXK2013-0014) in the context of the guidelines of the Jiangsu Province Animal Regulations (Government Decree No. 45). During feeding and study, health status of mice was monitored twice a day and recorded the clinical signs (ruffled hair coat, hunched posture, and diarrhea). If animals displayed clinical signs of illness, they were euthanized by cervical dislocation. Streptomycin-treated Balb/c mice were used to orally feed 3 × 10^8^ CFU live EHEC O157:H7 strain 86-24 each week for 4 weeks. Mice were bled 2 weeks after the last booster. Sera were stored in a -80°C freezer.

### Development Indirect ELISA for Detection Z3276 Expression *in Vivo* and *in Vitro*

For detection Z3276 expression *in vivo*, ELISAs were performed in 96-well plates (Costar, United States) coated with recombinant Z3276 antigen (0.6 μg/mL) and incubated at 4°C overnight. Plates were washed and blocked with 1% bovine serum albumin (BSA) in phosphate buffered saline containing 0.05% Tween 20 (PBST). The mouse negative sera and anti-EHEC O157:H7 sera were, respectively, added to antigen-coated wells and incubated at 37°C for 1 h. Sera were removed prior to adding goat anti-mouse IgG-HRP (1/5000 in PBST) (Boster, Wuhan, China) for 45 min at 37°C. The substrate solution TMBS (Sigma) was added into the washed wells. After 5 min, 2 M H_2_SO_4_ was used to stop the ELISA followed by read absorbance at 450 nm.

For detecting Z3276 expression *in vitro*, the method was the same as above, with some differences: (1) For coating antigen preparation, the EHEC O157:H7 strain 86-24 was cultured in TSB medium for 6 h, and inactivated with 0.3% formaldehyde solution at 37°C for 24 h until no live bacteria remained. The inactivated bacteria were washed two times with 0.01 M PBS (pH = 7.4) and used as antigen for coating plate; (2) the coating concentration was 10^8^ CFU/mL; (3) The primary antibody was the recombinant Z3276 protein polyclonal antibody from rabbits.

### Construction of Δ*z3276* and the Complemented Strain

The mutant was generated using the suicide plasmid as described previously (Dozois et al., 2003). Briefly, DNA sequences flanking *z3276* were amplified from the chromosomal DNA of EHEC O157:H7 strain EDL933 by PCR with two pairs of specific primers (P1-F/R and P2-F/R) carrying *BamH* I/*EcoR* I and *Sal* I/*EcoR* I restriction enzyme sites, respectively (**Table 2**). A DNA fragment containing the gentamicin resistance-encoding gene cassette was also obtained by PCR amplification from FastBac plasmid with primers (P3-F/R) carrying *EcoR* I restriction enzyme sites (**Table 1**). Gel-purified PCR products were cloned into pMEG375 to construct a suicide plasmid designated pMEG375-*z3276*FRGm, which was then transformed into *E. coli* strain CC118, named CC118-1. The positive plasmid pMEG375-*z3276*FRGm was transformed into a λpir lysogen of strain SM10, named SM10-1. The SM10-1 was hybridized with 86-24 strain on agar plate to produce Δ*z3276* mutant by homologous recombination. The Δ*z3276* was confirmed by PCR screening with *z3276*-specific primers (P4-F/R) (**Table 2**), DNA sequencing and Western blot analysis. The full-length *z3276* gene was amplified by PCR (P5-F/R) from EDL933 genomic DNA to generate a complementation strain cΔ*z3276*. The PCR primers used are listed in **Table 2**. The amplified 1344-bp DNA fragment of the *z3276* gene was inserted into the pMD19-T vector, and the recombinant plasmid was confirmed by DNA sequencing and restriction enzyme digestion. The positive pMD19-T-*z3276* was introduced into the Δ*z3276* strain to overexpress the Z3276 protein for reconfirmation of its function.

**Table 2:** Primers used in this study.

Primer	Nucleotide sequences (5′–3′)	Restriction sites	PCR product (bp)
P1-F/R	taaggatcctcgggaatttctaag	*BamH* I	343
	ggggaattcgtgaaatacgattaa	*EcoR* I	
P2-F/R	ggggaattcatttctgaacatcat	*EcoR* I	407
	taagtcgacgacggactgacacctta	*Sal* I	
P3-F/R	gaattcaccgtggaaacgcatgaag	*EcoR* I	811
	gaattcacggcttgaacgaattgtt	*EcoR* I	
P4-F/R	tttagtaaaagtgtcgtgtttact	/	898
	aatcgtatttcacgttgattaatg	/	
P5-F/R	atgatgttcagaaatagaatatta	/	1035
	ttaatcgtatttcacgttgattaa	/	
P6-F/R	cgcggatcctttagtaaaagtgtc	*BamH* I	916
	ggcaagcttaatcgtatttcacgtt	*Hind* III	

### Crystal Violet Biofilm Assay

A static biofilm formation assay was performed in 96-well polystyrene plates as previously reported (Lourenço et al., 2013; Eberly et al., 2017). Briefly, overnight cultures were diluted to an OD_600_ of 0.05 in TSB medium and Minca medium, respectively (300 μL) and incubated for 10 h without shaking at 37°C. After the cell density had been measured (turbidity at 630 nm), the plates were washed three times with PBS to remove all planktonic cells, and then 300 μL of 0.1% crystal violet was added to each well. After 20 min at room temperature, the microplate was emptied and washed three times with PBS. Next, 300 μL of 95% ethanol was added to resolve the stained biofilm cells. The total number of biofilm cells (absorbance at 570 nm) was measured and the total biofilm (OD_570_) was normalized with cell growth (OD_630_).

### Bacterial Adherence to Intestinal and Non-intestinal Cells

Cell adherence assays were performed as previously described with some modifications (Segura and Gottschalk, 2002). Bacteria were centrifuged, washed twice with PBS, and resuspended at 10^7^ CFU/mL in RPMI 1640 medium without antibiotics. We employed human laryngeal carcinoma cell line HEp-2 cells and porcine neonatal jejunal epithelial cell line IPEC-J2 cells. Monolayers of these cells lines grown in 24-well plates were infected at a multiplicity of infection of 10 bacteria per cell. The plates were centrifuged at 800 ×*g* for 10 min and incubated in RPMI 1640 medium with 2% newborn calf serum for 3 h at 37°C with 5% CO_2_. After washing three times with PBS and digesting with a mixture of 0.25% tryptase and 0.02% EDTA, adherent bacteria were plated onto sorbitol MacConkey (SMAC) agar plates to count the CFU. All assays were performed in triplicate and repeated three times. The results were expressed as the adherence rate relative to that of the WT set as 100%.

### Adherence Inhibition Assays

Anti-Z3276 polyclonal antiserum, for which the sensitivity and specificity were demonstrated by western blot analysis, was made in our laboratory. Briefly, we constructed a pcold I-*z3276* plasmid that could express abundant Z3276 protein in BL21 (DE3) cells. Purified Z3276 protein was used to immunize rabbits. Prior to adding onto the cell monolayer, 10 μL of the bacterial inoculum were incubated with 1:10, 1:50, and 1:100 dilutions of the anti-Z3276 and pre-immune bacterial suspensions at 37°C with gentle agitation. The number of adherent bacteria per cell in each sample was determined by plating onto SMAC plates at 37°C.

### Invasion Assay

The procedure for the invasion assay was the same as that for the adherence assay except the one ceftriaxone (10 μg/mL) and kanamycin (100 μg/mL) were added and incubated with the cells for 2 h to kill any extracellular bacteria before digestion. To confirm that all extracellular bacteria were killed by the antibiotic, 200 μL of the final PBS wash solution was plated on SMAC agar. Invasion frequencies were calculated as the number of bacteria surviving incubation with antibiotics divided by the total number of bacteria present in the absence of the antibiotic. All assays were performed in triplicate and repeated three times. The results were expressed as the invasion rate relative to that of the WT set as 100%.

### *In Vivo* Experiments

We used a streptomycin-treated mouse model to investigate the colonization ability of the strains. BALB/c mice were bought from experimental animal center of Yangzhou University (Yangzhou, China), housed in microisolator cages, provided with food and water *ad libitum*. To the study, the health status of mice was monitored twice a day and recorded the clinical signs (ruffled hair coat, hunched posture, and diarrhea). Thirty 6-week-old female BALB/c mice (*n* = 30) were divided into three groups and orally challenged, after anesthetized by inhaling diethyl ether, with WT, Δ*z3276*, and cΔ*z3276* strains at a dose of 10^10^ CFU in PBS, respectively. Mice infected with sterile PBS were used as controls. Fecal samples were taken on alternate days to monitor for shedding over 2 weeks.

### Twitching Motility Assay

Motility assays were performed as described previously with some modifications (Mattick, 2002). Briefly, 100 μL of an overnight culture was re-inoculated into 5 mL of sterile TSB and incubated at 37°C without shaking until logarithmic phase. One microliter of each bacterial culture was dropped onto semi-solid agar plates, which were incubated for 20 h at 37°C before analysis. Motility was observed by measuring the diameter of the motility halo. Non-motile strains grew only at the site of inoculation.

### Statistical Analysis

Where appropriate, data were expressed as the mean ± SEM. The difference from two groups was analyzed using the Student’s *t*-test and GraphPad Prism 5 software.

## Results

### Phylogeny Analysis of Z2376 Protein

**FIGURE 1:**
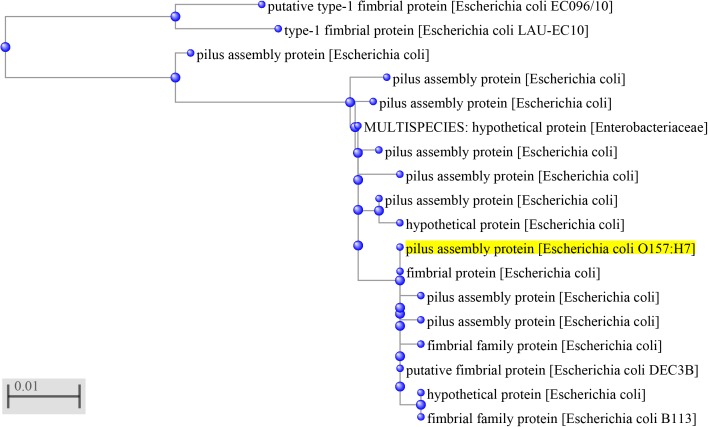
Phylogenetic relationships between Z3276 protein and other fimbrial proteins. The phylogenetic tree was generated by BLAST in GenBank with Z3276 protein (yellow) encoded by the entire *z3276 gene* from EHEC O157:H7 genome. The scale bar represents 0.01 nucleotide substitutions per site.

The BLAST analysis indicates that Z3276 amino-acid sequence has high identity between 91 and 100% to fimbrial proteins in GenBank (**Figure 1**), suggesting a conserved fimbrial domain in the Z3276 protein.

### Protein Expression and Purification

Recombinant plasmid pCold I-*z3276* was sequenced to indicate that *z3276* has 100% identity to reference sequences (GenBank CP015855.1) using Genscript Biotechnology, Co., Ltd. (Nanjing, China). Recombinant bacteria BL21/pCold I-*z3276* was induced by IPTG. SDS-PAGE showed that recombinant Z3276 (33 kDa) was successfully expressed with 35% proportion to whole bacterial protein in contrast to naïve bacteria.

### Development of pAb Against Z3276 and EHEC O157:H7

Anti-Z3276 was successfully prepared from rabbits 229 and 230, and the titers were 2.6 × 10^6^ and 7.9 × 10^5^, respectively, using indirect ELISA. Anti-EHEC O157:H7 was successfully prepared from 80% (4/5) mice, and the highest titer and the lowest titer were 9 × 10^4^ and 3 × 10^3^, respectively.

### Indirect ELISA for Detection Z3276 Expression *in Vivo* and *in Vitro*

The ELISA plate coated with Z3276 was used to incubate with anti-EHEC O157:H7 sera and negative sera. The anti-EHEC O157:H7 sera gave an OD450 value greater than 1.0, compared with lower OD450 values from negative sera (**Table 3**). The detection data indicated Z3276 protein could express *in vivo* and perhaps on the bacterial surface.

**Table 3:** Z3276 expression *in vivo* using indirect ELISA.

	1:200	1:400	1:800	1:1600
Negative mouse sera	0.142	0.117	0.085	0.051
Anti-EHEC O157:H7 sera	1.445	1.201	0.923	0.631

The ELISA plate coated with the inactivated EHEC O157:H7 was used to incubation with anti-Z3276 sera and negative sera. The anti-EHEC O157:H7 sera gave OD450 value was greater than 1.0, compared with lower OD450 value from negative sera (**Table 4**). The detection data indicated Z3276 protein could express under the experimental conditions.

**Table 4:** Z3276 expression *in vitro* using indirect ELISA.

	1:200	1:400	1:800	1:1600
Negative rabbit sera	0.191	0.138	0.056	0.029
Anti-Z3276 sera	2.282	1.931	1.602	1.242

### Construction of a *z3276*-Defective Mutant

**FIGURE 2:**
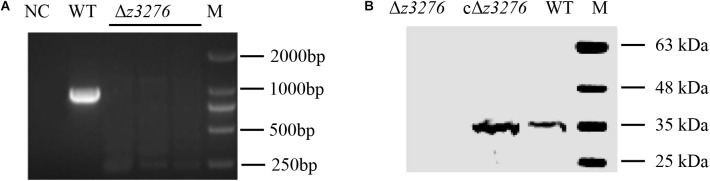
Confirmation of the Δ*z3276* in EHEC O157:H7 strain 86-24. **(A)** Confirmation of the Δ*z3276* by PCR using primers P4-F/R. M, 2000-bp marker. The predicted PCR product size is 898 bp. **(B)** Confirmation of the Δ*z3276* by Western blot analysis. The predicted protein size is 33 kDa.

The Δ*z3276* mutant was constructed by homologous recombination and was confirmed by PCR (**Figure 2A**) and Western blot analysis (**Figure 2B**). PCR results confirmed that the Δ*z3276* mutant was negative for *z3276* gene, and no reaction was detected between Δ*z3276* and anti-Z3276 serum by the Western blot assay. In term of growth stability, the Δ*z3276* and cΔ*z3276* strains could be subcultured very well at least for 20 passages.

### Z3276 Contributes to Biofilm Formation

**FIGURE 3:**
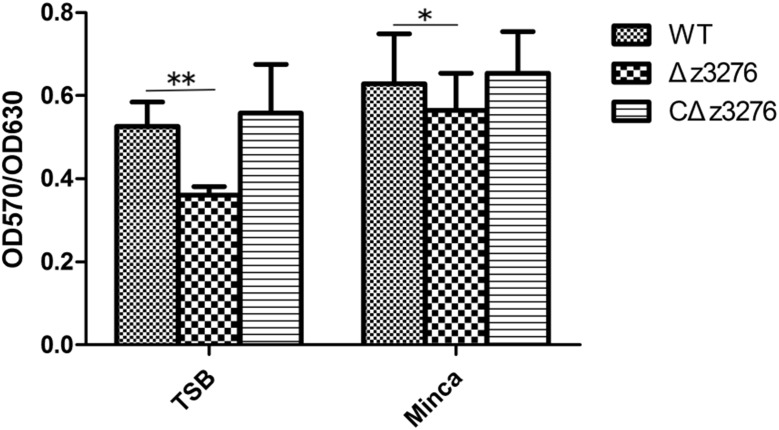
Δ*z3276* decreases biofilm formation in TSB medium and modified Minca medium at 37°C without shaking after 10 h in 96-well plates. Total biofilm formation (OD_570_) was normalized by bacterial growth (OD_630_). The experiment was performed in triplicate, and data represent the mean ± SEM of three independent experiments. ^∗^Indicates significance at *P* < 0.05; ^∗∗^Indicates extreme significance at *P* < 0.01.

To test whether Z3276 was important for biofilm formation by EHEC O157:H7 on abiotic substrata, we compared the abilities of the WT, Δ*z3276*, and cΔ*z3276* strains to form biofilms in 96-well polystyrene plates. The results indicated that Δ*z3276* was reduced in its ability to form biofilms compared with the WT strain and complemented strains (**Figure 3**) regardless of culture media used. Mutant strains lost more abilities to form biofilms when cultured in TSB medium (*P* < 0.01) than in Minca medium (*P* < 0.05). Complemented strains regained stronger biofilm-forming abilities in a greater extent than WT strains had (*P* > 0.05).

### Anti-Z3276 Serum Blocked the Adherence of the WT Strain to Both HEp-2 and IPEC-J2 Cells

**FIGURE 4:**
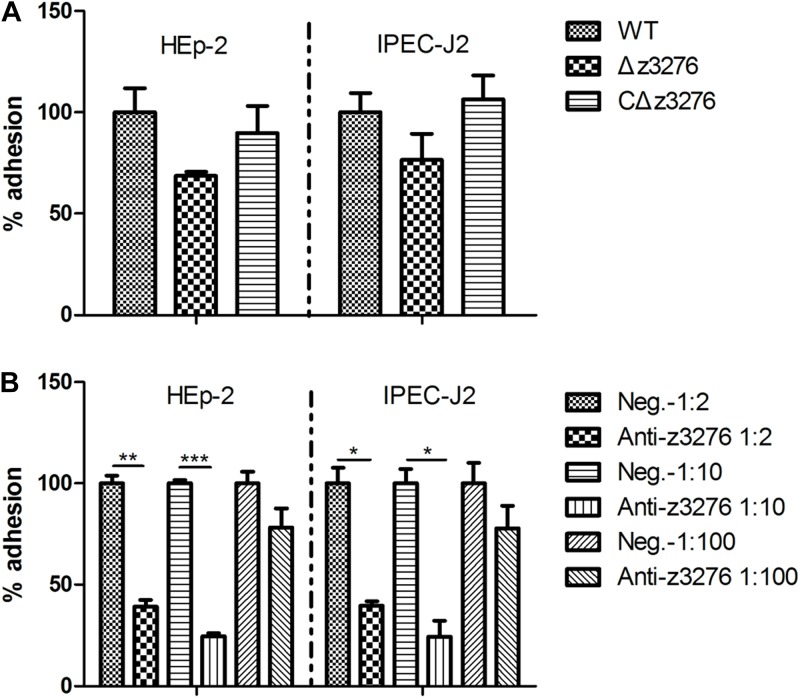
Interaction of the WT and Δ*z3276* with HEp-2 and IPEC-J2 cells. **(A)** The Δ*z3276* mutant showed no change of adherence to either cell line compared with the parental strain. **(B)** Adherence of WT EHEC O157:H7 strain to HEp-2 and IPEC-J2 cells reduced after pre-incubation with blocking antibody (1:2 and 1:10 dilution). The WT strain adhesion index was assumed to be 100%. The results shown are the means + SEM of three independent experiments. ^∗^Indicates significance at *P* < 0.05, ^∗∗^ and ^∗∗∗^Indicate extreme significance at *P* < 0.01 and *P* < 0.001.

No significant difference was detected in the adherence ability of the Δ*z3276* mutant compared with the WT strain to both HEp-2 and IPEC-J2 cells (*P* > 0.05, *P* > 0.05, respectively) (**Figure 4A**). However, an obvious reduction was observed in the adherence of the WT strain to HEp-2 and IPEC-J2 cells after incubation with an anti-Z3276 antibody at a 1:2 dilution (antibody titer = 1:12,800) (*P* < 0.05, *P* < 0.05, respectively), and a 1:10 dilution (*P* < 0.05, *P* < 0.05, respectively) (**Figure 4B**), but not at a 1:100 dilution (*P* > 0.05, *P* > 0.05, respectively) (**Figure 4B**).

### Expression of Z3276 Could Trigger the Invasion of IPEC-J2 Cells

**FIGURE 5:**
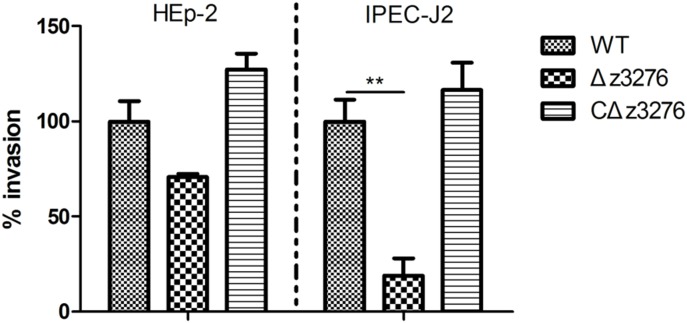
The ability of Δ*z3276* to invade IPEC-J2 cells was decreased compared with the wild-type strain. Ceftriaxone (10 μg/mL) and kanamycin (100 μg/mL) were added to ensure that only intracellular bacteria were obtained. The WT strain adhesion index was assumed to be 100%. The results shown are the means ± SEM of three independent experiments. ^∗∗^Indicates extreme significance at *P* < 0.01.

Invasion of host cells is a crucial step in bacterial pathogenesis. Compared with the WT strain, we found no change in the rate of invasion of HEp-2 cells for the Δ*z3276* mutant (*P* > 0.05), whereas an 81% reduction in invasion of IPEC-J2 cells was observed for the Δ*z3276* mutant (*P* < 0.05) (**Figure 5**). These results indicated that *z3276* may be involved in the pathogenicity of EHEC O157:H7 in specific cell types.

### Δ*z3276* Mutant Showed Decreased Colonization in Mice

**FIGURE 6:**
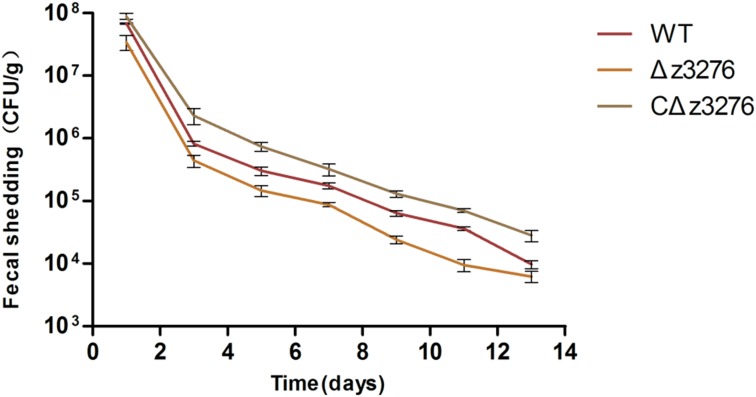
Δ*z3276* showed shortened and lower fecal shedding in mice. Mice were orally challenged with strains WT, Δ*z3276*, cΔ*z3276*, or PBS. Fecal samples were collected on alternate days over a 2-week period. The CFUs in each sample were determined by plating onto SMAC plates at 37°C. The results shown are the averages of five mice per group.

After treating mice with streptomycin (5 g/L for the first 3 days and then 0.5 g/L) and mitomycin C (2.5 mg/kg) to enhance their sensitivity (Zhang et al., 2011, 2014), we challenged with the WT and Δ*z3276* via oral–gastric inoculation. The results revealed shortened and lower fecal shedding for the Δ*z3276* compared with the WT strain (*P* < 0.05) (**Figure 6**), which indicated that Z3276 protein may be involved in colonization *in vivo*.

### Motility of the 86-24 WT and the Δ*z3276* Mutant Strains

**FIGURE 7:**
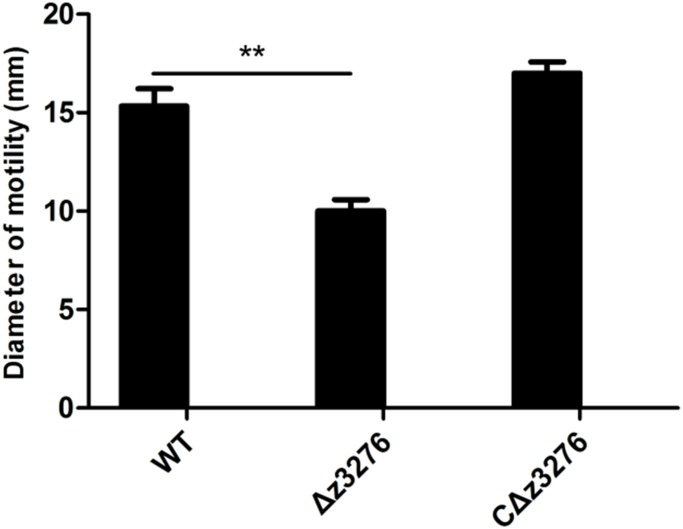
Swimming motility diameter of WT, Δ*z3276*, and cΔ*z3276*. Data represent the means ± SEM of three separate experiments. ^∗∗^Indicates extreme significance at *P* < 0.01.

Twitching motility is a phenomenon associated with virulence in many Gram-negative bacteria (Mattick, 2002), and is mediated by the retraction and extension of flexible pili by bacteria growing on a semi-solid surface. In this study, both the WT and Δ*z3276* were motile on agar. A 15-mm twitching motility zone on the semi-solid agar surface was observed for the WT, compared with a 50% reduction in the zone for the Δ*z3276* mutant strain (*P* < 0.05) (**Figure 7**).

## Discussion

EHEC is a zoonotic pathogen, of which O157:H7 is the most important serotype responsible for a number of outbreaks in animals, poultry, and humans worldwide, leading to serious public health concern. The locus of enterocyte Effacement Island, a prophage producing two Shiga toxins and a 60 MDa virulence plasmid pO157 are considered the major determinants of EHEC O157:H7 pathogenesis (Sjogren et al., 1994; Schmedt et al., 1996). EspP was recently recognized as a new member of the serine protease autotransporters of Enterobacteriaceae family and, contributing to increased hemorrhaging into the intestinal tract (Khan et al., 2009) and evasion from immune system-mediated elimination (Orth et al., 2010). In addition to above-mentioned defining virulence factors of EHEC O157:H7, there are other factors that may also contribute to its pathogenicity.

Increasing evidence suggests that fimbrial play an important role in the initial stages of EHEC O157:H7 infections (Xicohtencatl-Cortes et al., 2007). Martinez and coworkers showed that the tip adhesin Fim H of type 1 fimbrial was sufficient to trigger invasion of uropathogenic *E. coli* into bladder epithelial cells (Martinez et al., 2000). However, the potential role of type 1 fimbrial in the pathogenicity of EHEC O157:H7 has not been reported, due to a nucleotide deletion and mutation in the *fim* operon encoding type 1 fimbrial (Li et al., 1997). Among the complete genome sequences available in current databases, the *z3276* gene was only detected in EHEC O157:H7 (Li and Chen, 2012), and its amino acid sequence showed high homology with other *E. coli* type I fimbrial. Therefore, it is possible that *z3276*-encoding protein is a compensatory mechanism for type I fimbrial.

Biofilms help bacteria colonize inert surfaces, whilst protecting the bacterial cells from the host immune defense system as well as from antibiotic drugs (Gocer et al., 2017; Russell et al., 2018). In this study, an obvious reduction in biofilm formation was observed with a Δ*z3276* mutant compared with the WT strain in modified Minca medium and TSB, but no change was detected in Mueller–Hinton broth (MHB), brain heart infusion broth (BHI) or LB broth, suggesting that Z3276 production *in vivo* may be modulated by the composition of the medium and other conditions. *Candida albicans* biofilms are highly resistant to the actions of clinically important antifungal agents due to major multidrug efflux pumps encoded by *C*. *albicans* and the important role of biofilms in the drug resistance of planktonic cells (Cao et al., 2005). Moreover, *E. coli* biofilm bacteria showed weaker inflammatory responses and enhanced resistance to some antimicrobial peptides, and the increased *in vivo* survival of biofilm bacteria in a clinically relevant model of catheter infection has been reported (Lloyd et al., 2012; Chalabaev et al., 2014). Therefore, we can speculate that the *z3276* gene in EHEC may confer the bacterium with resistance against antibiotics, thereby increasing its survival time in the host or the environment, which is critical to the transmission and infection.

Adherence is the first step in bacterial invasion. In the present study, we explored whether Z3276 plays a role in the adherence between the bacteria and cultured intestinal (IPEC-J2 cells) and non-intestinal (HEp-2 cells) epithelial cells. Qualitative analysis from IPEC-J2 cells infected with Δ*z3276* and WT showed no significant difference in the levels of adherence, with values of 1.2 × 10^5^ CFU/mL and 1.6 × 10^5^ CFU/mL, respectively. The same result was observed with HEp-2 cells. One possibility is that HEp-2 cells and IPEC-J2 cells do not possess a suitable receptor to interact with *z3276*-encoding protein, however, *z3276*-encoding protein may contribute to the adherence to other cell types or to abiotic surfaces. For instance, the Δ*z3276* and its parental strain showed similar levels of invasion of HEp-2 cells. In contrast, the Δ*z3276* mutant showed 81% decrease in invasion ability to IPEC-J2 cells compared with the WT strain. Thus, we speculate that there might be certain moieties that can recognize Z3276 specifically and “carry” the bacterium into cells.

A mouse model was used to evaluate the colonization ability of the Δ*z3276* and the WT strains *in vivo*. The data of the animal study demonstrated that the Δ*z3276*-infected mice rendered the animals lower bacterial counts and higher clearance efficiency, indicating that the ability of Δ*z3276* mutant to colonize the host and thereby survive in the host was impaired. This may be partially due to the reduced capacity to invade epithelial cells. The z3276 gene in EHEC may encode a fimbrial protein, or termed as tip adhesin (in the distal end of the pili), which has been previously reported to mediate the specific attachment to tissues or surfaces (Lindberg et al., 1987; Kaper et al., 2004; Low et al., 2006; Gu, 2017).

Besides as a colonization factor, Z3276 was also shown here to be involved in the twitching motility of EHEC O157:H7. This property may contribute to the pathogenicity of EHEC O157:H7, as it has been demonstrated that flagellum/fimbrial-mediated motility is essential for enhancing pathogen–host interactions and for promoting the subsequent adherence and colonization of several other Gram-negative pathogens (La Ragione et al., 2000; Wright et al., 2007; Mahajan et al., 2009).

In summary, this study confirmed that the *z3276* gene in EHEC O157:H7 encodes multifunctional structures with properties that may contribute to host colonization and bacterial survival in the environment.

## Author Contributions

XZ and KH conceived and designed the experiments. BZ and XS performed the experiments. HF analyzed the data. BZ and XZ wrote the paper.

## Conflict of Interest Statement

The authors declare that the research was conducted in the absence of any commercial or financial relationships that could be construed as a potential conflict of interest.
